# Robot-assisted laparoscopic resection of giant pelvic solitary fibrous tumor: a case report with literature review

**DOI:** 10.3389/fonc.2026.1887824

**Published:** 2026-07-01

**Authors:** Binbin Wang, Gengchen Huang, Wei Wei, Tie Mao, Zihan Gao, Yutao Ma, Yiming Gu

**Affiliations:** 1 Department of Urology II, The First Hospital of Jilin University, Changchun, China; 2 Department of Operating Room I, The First Hospital of Jilin University, Changchun, China

**Keywords:** diagnosis, giant tumor, pelvic neoplasms, robot-assisted laparoscopy, solitary fibrous tumor, surgical treatment

## Abstract

Solitary fibrous tumor (SFT) is a rare mesenchymal neoplasm with a low incidence, and pelvic SFT is extremely uncommon. Due to its insidious onset and nonspecific imaging features, preoperative diagnosis is difficult. We reported a 34-year-old male patient with a giant pelvic SFT found incidentally during physical examination, complicated with left hydronephrosis and hydroureter. Preoperative imaging revealed a large heterogeneous enhanced mass in the pelvic cavity. Robot-assisted laparoscopic resection of the tumor was performed successfully, accompanied by simultaneous ureteral reimplantation due to severe adhesion between the tumor and the left ureter. The operation was completed with R0 resection. Postoperative pathology and immunohistochemistry (STAT6+, CD34+, Bcl-2+) confirmed the diagnosis of SFT. The patient recovered smoothly and was followed up for 12 months without tumor recurrence or complications. For selected patients with giant pelvic SFT, robot-assisted laparoscopic surgery appears to be a viable treatment option. This technique facilitates meticulous dissection and yields promising short-term clinical outcomes. However, long-term follow-up is required due to the potential for late recurrence of SFT.

## 1 Introduction

Solitary fibrous tumor (SFT) is a rare mesenchymal spindle-cell neoplasm with an annual global incidence of approximately 1–2 per million people (1–3). SFT affects males and females equally, occurs predominantly in adults, and peaks at 40–70 years of age (4). SFT can arise in various anatomical sites, most commonly in the pleura, accounting for about 30% of all cases. Other frequent locations include the meninges (27%), the abdominopelvic cavity (20%), the trunk (10%), the extremities (8%), and the head and neck (5%) (5, 6). However, SFT accounts for less than 2% of all soft-tissue tumors (7).

The pelvis is an extrapleural site of SFT, representing only 6% of all SFTs (8). Pelvic SFT usually originates from deep tissues and may involve the sigmoid colon, rectum, prostate, bladder, sacral canal, perineum, ischiorectal fossa, and ischioanal fossa (9). Due to the spacious pelvic cavity and insidious growth, pelvic SFT is usually asymptomatic in the early stage. It often grows to a large size before causing non-specific compressive symptoms, mainly including abdominal pain, dysuria, urinary retention, numbness and weakness of the lower extremities secondary to nerve compression, constipation, and painful micturition (9). Owing to its low incidence, limited clinical awareness, and overlapping imaging and clinical features with other soft-tissue tumors, preoperative diagnosis of SFT is difficult with a high misdiagnosis rate, posing substantial challenges in clinical diagnosis and management (3, 10).

The diagnosis of SFT relies on histopathological and immunohistochemical examinations. Histologically, most SFTs show a patternless or storiform architecture, characterized by alternating hypercellular and hypocellular areas separated by fibrous stroma, accompanied by hemangiopericytoma-like branching vessels (11). Immunohistochemically, nuclear STAT6 positivity is the most specific diagnostic marker of SFT, with a sensitivity of 95.8% and specificity of 88.3%, representing the current gold standard for diagnosis (12). Nuclear STAT6 expression is driven by the NAB2–STAT6 fusion gene, which is detected in nearly all SFTs and serves as a highly specific molecular marker for this tumor (13).

Surgical resection with microscopically negative margins (R0 resection) is the standard treatment for SFT and effectively reduces the risk of local recurrence (4). However, giant pelvic SFT is difficult to manage due to its large volume, rich blood supply, and frequent dense adhesion to adjacent structures. Traditional open surgery is associated with substantial trauma, high bleeding risk, and slow postoperative recovery, whereas the narrow pelvic space limits conventional laparoscopy and demands high surgical proficiency (3). In contrast, Robot-assisted laparoscopic surgery offers ergonomic instrumentation, three-dimensional vision, and seven degrees of freedom of motion, allowing precise dissection in confined pelvic cavities, thereby reducing surgical difficulty, intraoperative bleeding, and postoperative complications (14–16). Nevertheless, reports on robot-assisted surgery for giant pelvic SFT remain scarce, and long-term follow-up data are lacking to verify its long-term efficacy and safety.

Herein, we report a case of giant pelvic SFT treated with robot-assisted laparoscopic resection. Combined with 1-year follow-up results and a literature review, we discuss the key diagnostic points, surgical strategies, and prognostic features of this rare tumor to provide a reference for clinical practice.

## 2 Case presentation

### 2.1 Patient history and preoperative examinations

A 34-year-old male patient was found to have a pelvic mass during physical examination 10 days ago. He was then admitted to our hospital for further evaluation and treatment. He denied any subjective discomfort, and his vital signs were stable on admission. Routine laboratory tests were within normal limits. Subsequently, pelvic magnetic resonance imaging (MRI) with plain, contrast-enhanced, and diffusion-weighted imaging (DWI) was performed. A lobulated mass was detected in the pelvic cavity, posterior to the bladder, superior to the prostate and seminal vesicles, and anterior to the rectum, measuring approximately 10.4 cm × 9.5 cm × 10.0 cm (craniocaudal × anteroposterior × transverse). The mass showed isointense to slightly hypointense signal on T1-weighted imaging (T1WI), heterogeneously slightly hyperintense signal on T2-weighted imaging (T2WI), heterogeneously slightly hyperintense signal on DWI, and isointense signal on apparent diffusion coefficient (ADC) mapping. Marked persistent heterogeneous enhancement was observed after contrast administration, with a relatively clear boundary between the lesion and surrounding tissues (**Figure 1**). The imaging findings were highly suggestive of SFT. Since MRI revealed abnormalities along the left ureteral course, contrast-enhanced multi-detector computed tomography (CT) of the urinary system was further performed. The results demonstrated left hydronephrosis and hydroureter of the proximal ureter, with an indistinct distal ureter. The pelvic mass showed marked heterogeneous enhancement in the arterial phase, with progressive, homogeneous enhancement in the venous and delayed phases. Compression and displacement of adjacent organs were also noted, indicating left hydronephrosis and hydroureter secondary to the pelvic mass (**Figure 2**).

**Figure 1:**
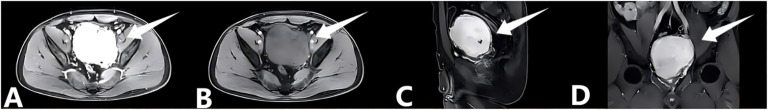
Pelvic MRI of solitary fibrous tumor. **(A)** Axial T2WI: heterogeneous hyperintense lobulated mass (arrow). **(B)** Axial T1WI: iso- to hypointense mass (white arrow) with clear borders. **(C)** Sagittal and **(D)** coronal contrast-enhanced T1WI: marked heterogeneous enhancement of the mass (white arrow).

**Figure 2:**
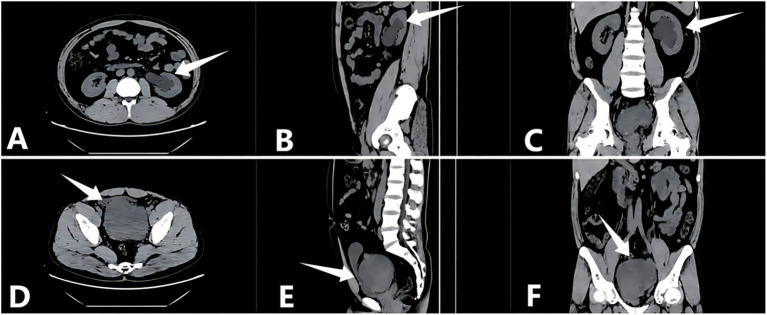
Contrast-enhanced CT findings of the pelvic solitary fibrous tumor. **(A–C)** Axial, sagittal, and coronal images show secondary left hydronephrosis and hydroureter (white arrows) caused by the pelvic mass. **(D–F)** Axial, sagittal, and coronal images demonstrate a large pelvic mass (white arrows) with mass effect.

### 2.2 Surgical strategy and procedure

Preoperative multidisciplinary team (MDT) consultation was performed to comprehensively evaluate the tumor size, anatomical location, and its relationship with adjacent tissues and organs. Surgical contraindications were excluded. Considering the patient’s preference, robot−assisted laparoscopic complete resection of the pelvic tumor was determined as the final surgical plan.

Surgical procedure: The patient was placed in the dorsal lithotomy position. Cystoscopy was performed first, revealing that the trigone of the bladder was significantly elevated by the tumor, with bilateral ureteral orifices clearly identified. Attempts were made to place bilateral ureteral stents, but stent placement on the left side failed. A 3−cm vertical midline incision was made below the umbilicus as the camera port, and pneumoperitoneum was established and maintained at 14 mmHg (14 cmH_2_O). The first and second working ports were created at the intersection of the bilateral pararectus abdominis muscles and the iliac crest line. The third and fourth working ports were placed at the junction of the bilateral pararectus abdominis muscles and 3 cm above the pubic symphysis. The robotic laparoscopic system was docked, and exploration revealed a large, bulging mass approximately 10 cm in diameter, located behind and below the bladder (**Figure 3A**). The pelvic peritoneum was opened to expose the tumor capsule, and careful dissection was performed along the capsule. The tumor was found to be extremely hypervascular, and strict hemostasis was maintained throughout the procedure. Dissection was carried out in the following sequence: right lateral wall (to protect the right ureter), superior wall (to protect the bladder), anterior wall (to protect the prostate and seminal vesicles), and finally left lateral and inferior walls (to protect the intestines). During the dissection of the left lateral wall, the left vas deferens was identified. Given its close association with the tumor, the left vas deferens was ligated and transected using Hem−o−lok clips (**Figure 3B**). Further dissection showed severe adhesion between the tumor and the left ureter, with a fragile ureteral wall. The left ureter was therefore transected, and simultaneous ureteroneocystostomy was planned (**Figures 3C, D**). After complete mobilization, the tumor was placed into an endopouch. The pneumoperitoneum pressure was reduced to 4 mmHg (4 cmH_2_O), and the pelvic cavity was inspected to confirm no active bleeding. Left ureteroneocystostomy was then performed (**Figure 3E**). After meticulous hemostasis, one pelvic drainage tube was placed through the fourth working port. The robotic instruments were undocked, and the pneumoperitoneum was released. The camera incision was extended to extract the specimen bag, and all incisions were closed layer by layer. The total operative time was 339 minutes. The estimated intraoperative blood loss was 1600 ml. The patient received a transfusion of 400 ml virus−inactivated frozen plasma and 4 U of leukocyte−depleted suspended red blood cells.

**Figure 3:**
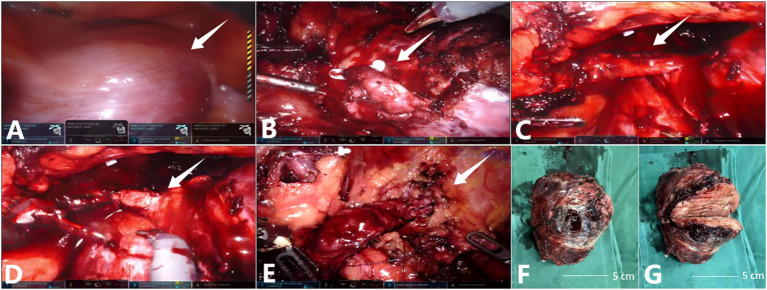
Intraoperative robotic laparoscopic views and gross appearance of the resected giant pelvic SFT. **(A)** Intraoperative view showing a large bulging pelvic mass located posterior and inferior to the urinary bladder (white arrow). **(B)** Ligation and transection of the left vas deferens using Hem-o-lok clips during tumor dissection (white arrow). **(C)** Severe adhesion between the tumor and the left ureter was observed intraoperatively (white arrow). **(D)** Clear urine outflow from the transected end of the left ureter (white arrow). **(E)** Completion of left ureteroneocystostomy (ureterovesical reimplantation; white arrow). **(F)** Gross appearance of the resected giant pelvic SFT (scale bar = 5 cm). **(G)** An incomplete section of the tumor showing its internal architecture (scale bar = 5 cm).

### 2.3 Postoperative recovery and pathological examination

After surgery, the patient received symptomatic treatments, including anti-infection therapy, hemostasis, fluid infusion, and nutritional support, with close monitoring of vital signs and pelvic drainage. Flatus passed on postoperative day 1, and the liquid diet was initiated. The drainage tube was removed on postoperative day 3 as the drainage volume was less than 20 ml. No complications, such as fever, abdominal pain, or bleeding, occurred. The patient recovered smoothly and was discharged on postoperative day 5 with normal blood routine and liver and renal function test results. Pathological examination was performed postoperatively: Gross examination showed a nodular pelvic mass measuring 10.2 cm × 8 cm × 7.5 cm, with a relatively smooth surface, tan-brown color, solid consistency, and a slightly tough cut surface (**Figure 4A**). Microscopically, the tumor was composed of spindle and ovoid cells with a disorganized arrangement, embedded in abundant collagenous stroma, and characteristic staghorn-like thin-walled vessels were visible (**Figure 4B**). The tumor cells showed no significant atypia, and the mitotic count <4/10 high-power fields (HPF). A focal hemorrhage was identified in the tumor. Most of the tumor margin was well-defined, with focal invasion into adjacent tissues. No tumor involvement was found in the spermatic cord or the surgical margin. Immunohistochemical staining results were as follows: Ki-67 (+, 5%), STAT6 (+), CD34 (+), Bcl-2 (+), Vimentin (+), SMA (focal +), P16 (focal +); CD117 (−), Dog-1 (−), Desmin (−), S-100 (−), SOX10 (−), β-catenin (−), CK-pan (−), MUC-4 (−), ALK (−), MDM2 (−), CDK4 (−), H-caldesmon (−). The pathological diagnosis was SFT.

**Figure 4:**
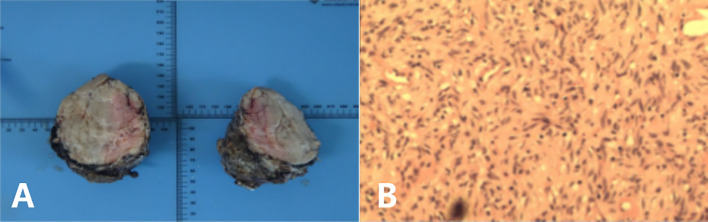
Pathological features of pelvic SFT. **(A)** Gross photograph of the resected tumor. **(B)** Photomicrograph (H&E stain, ×100) showing spindle cell morphology with characteristic hemangiopericytoma-like vessels.

### 2.4 Follow-up results

The patient was followed up regularly after discharge according to the scheduled protocol. Follow-up time points included 1, 3, 6, and 12 months after surgery. The follow-up items included physical examination, laboratory tests, and radiological evaluation. At 1 month postoperatively, a plain abdominal film (KUB) showed that the bilateral double-J ureteral stents were in good position. Cystoscopic removal of bilateral ureteral stents was performed at the same time. During the 1-year follow-up period, the patient remained free of symptoms such as abdominal pain, abdominal distension, dysuria, or defecation disorder. His diet, sleep, and physical performance all returned to normal, with a good quality of life. All laboratory results were within normal ranges. Imaging examination showed no evidence of local recurrence or distant metastasis of the pelvic tumor. The anatomical structure of the pelvic organs was restored, and left hydronephrosis and hydroureter were relieved (**Figure 5**).

**Figure 5:**
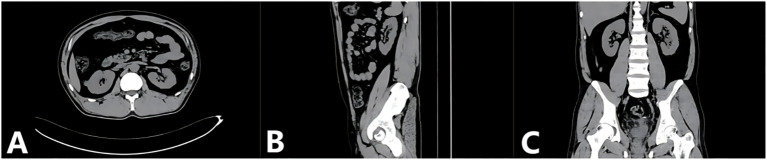
6-month follow-up CT. **(A–C)** Axial, sagittal, and coronal views show resolution of left hydronephrosis and no evidence of pelvic tumor recurrence.

## 3 Discussion

Pelvic SFT is a rare mesenchymal neoplasm with an insidious onset and no specific early symptoms. It is usually detected incidentally during physical examination or when the tumor enlarges and causes compressive symptoms. It is easily misdiagnosed as common pelvic tumors such as ovarian tumor (17), uterine leiomyoma (18), or intestinal tumor (9, 19), with a preoperative misdiagnosis rate of 10%–20% (3). SFT is composed of spindle cells producing fibrous collagen and myxoid materials and is characterized by abundant blood supply (20). On CT images, SFT appears as a well−circumscribed hypervascular mass, and large tumors may exert mass effect on adjacent structures (20). On contrast−enhanced CT and MRI, SFT shows marked enhancement with heterogeneous patterns: hypervascular regions show early intense enhancement, cellular regions show moderate enhancement, and areas with necrosis, cystic degeneration, or myxoid change show no enhancement (21). On MRI, SFT is usually isointense on T1WI and shows variable signal intensity on T2WI; low signal intensity on T2WI often indicates collagen-rich stroma (20, 22). Lesions with such imaging features require differential diagnosis from other hypervascular or fibrous-rich tumors, including leiomyosarcoma, neurogenic tumor, pheochromocytoma, lymphoma, desmoid tumor, malignant fibrous histiocytoma, mesothelioma, and fibroma (21). Therefore, imaging findings of SFT are nonspecific, and a definite diagnosis relies on pathological examination and immunohistochemistry after complete resection. STAT6 is the most valuable diagnostic marker for SFT, and positive CD34, BCL−2, and CD99 can also assist in diagnosis (12, 23). In the present case, preoperative imaging revealed a giant solid pelvic mass, and SFT was confirmed by postoperative pathology and immunohistochemistry (STAT6+, CD34+, Bcl−2+), consistent with previous reports. This suggests that preoperative imaging, combined with postoperative pathology and immunohistochemistry, is crucial for diagnosing pelvic SFT. Regarding treatment, R0 resection is the first−line therapy for SFT and is critical for reducing recurrence (24). For giant pelvic SFT as in this case, the core of surgery is to achieve complete tumor resection while maximally protecting vital adjacent organs and vessels to minimize intraoperative bleeding and complications. Although traditional open surgery can achieve complete resection, it is associated with major trauma, heavy bleeding, slow recovery, and high risks of wound infection and intestinal adhesion. Laparoscopic resection is feasible for small pelvic SFT but challenging for large tumors due to the narrow pelvic space and poor surgical exposure (3). Robot−assisted laparoscopic surgery offers high−precision instruments, clear three−dimensional vision, tremor−free manipulation, and ergonomic design, which overcome the limitations of conventional laparoscopy (25). Compared with conventional laparoscopy, robotic surgery can shorten hospital stay and reduce the conversion rate to open surgery (15). Such strengths make it an ideal option for tumor resection in complex sites such as the pelvis, enabling precise dissection of adhesive tissues, reducing bleeding and complications, and promoting postoperative recovery. However, our case had an intraoperative blood loss of 1600 mL. This large blood volume was not a defect of the robotic surgical approach, but was closely related to the pathological and anatomical features of this giant pelvic SFT. As a typical mesenchymal neoplasm, pelvic SFT is universally characterized by abundant blood supply (23); this tumor measured over 10 cm in diameter, with a dense network of feeding vessels within and on its surface. In addition, long-term tumor compression led to severe inflammatory adhesion between the lesion and the left ureter, pelvic floor soft tissues and surrounding organs. The separation of extensive adhesions in the confined pelvic cavity led to widespread capillary oozing, which was difficult to control completely with hemostatic measures alone. Despite careful dissection, massive bleeding was unavoidable. Adequate preoperative blood preparation and timely transfusion were performed, but this still increased the surgical risk to some extent. Therefore, for such hypervascular giant pelvic SFT, proactive preoperative strategies can be adopted, such as identifying tumor feeding vessels by angiography and performing transcatheter arterial embolization (TAE) to facilitate complete resection and reduce or avoid fatal intraoperative bleeding (26). More importantly, this case required not only total tumor resection, but also synchronous ureteral transection and ureteroneocystostomy due to irreversible ureteral injury. Robot-assisted laparoscopy, with its superior spatial resolution and multi-angle operating instruments, created favorable conditions for both tumor dissection and urinary tract reconstruction, and effectively reduced the risk of iatrogenic injury to adjacent vital organs. From the perspectives of surgical safety, feasibility of combined operations, and postoperative rehabilitation, robotic laparoscopy was a reasonable and individualized choice for this patient.

We further compared our case with the recent study by Xie et al. (2025), focusing on robot-assisted laparoscopic resection of periprostatic SFT (27). Both studies explored robotic minimally invasive surgery for rare pelvic SFT, yet they differed significantly in tumor characteristics, surgical strategies and the management of associated complications. The lesion in Xie et al.’s report was a small periprostatic SFT measuring 5.0 cm that adhered to the prostatic fascia without compressing or adhering to the urinary tract. The surgery was designed to preserve the prostate and neurovascular bundle to protect the patient’s urinary and sexual function. In contrast, our case involved a giant pelvic SFT of 10.4 cm with rich vascularity, which led to left hydronephrosis and severe ureteral adhesion. Due to irreversible ureteral damage caused by long-term tumor compression, we completed R0 tumor resection combined with synchronous ureteroneocystostomy, adding urinary tract reconstruction procedures and increasing the overall surgical difficulty. Correspondingly, the perioperative protocols also varied: while the prior study required only routine functional monitoring after organ-preserving surgery, our case implemented targeted interventions, including preoperative blood preparation and attempted ureteral stenting, as well as continuous postoperative surveillance for urinary tract patency. The present study’s core innovation is the summary of surgical skills and the full perioperative experience for robot-assisted laparoscopy applied to giant pelvic SFT complicated by urinary tract obstruction and severe adhesions. Our findings verify the feasibility of robotic surgery for such complex large pelvic tumors, and together with the 12-month follow-up results, offer a novel reference for the individualized diagnosis and surgical management of complex pelvic SFT.

The patient achieved R0 resection and was followed up for 1 year without tumor recurrence, with a good quality of life. However, studies have shown that patients with SFT undergoing R0 resection have 5−year survival rates of 59%–100% and 10−year survival rates of 40%–89% (28). SFT exhibits substantial biological heterogeneity. Most extrapleural SFTs follow a benign clinical course, yet 10%–15% of cases carry a risk of local recurrence or malignant transformation (29). Because the prognosis of SFT is unpredictable, even histologically benign lesions may recur late, 10–20 years after surgery (10, 30, 31). Therefore, long−term and standardized follow−up is essential for all patients with SFT (32). Valid risk stratification models provide important guidance for individualized treatment and follow−up strategies (**Table 1**). The original Demicco model was the first to establish a three−tiered risk stratification system based on age, tumor size, and mitotic count, laying the foundation for risk assessment in SFT (33). The SalasOS model further simplified the indicators to age and mitotic count, thereby enabling rapid clinical stratification (34). The modified Demicco model (mDemicco) added tumor necrosis as a variable and has been validated in multi−institutional studies with stable predictive performance, making it one of the most widely used tools in clinical practice (35). The G−score model innovatively incorporated sex as a parameter and is more advantageous for predicting late recurrence (36). Recently, Zhang et al. (2023) proposed an integrated risk model combining histopathology, immunohistochemistry, and molecular biomarkers. In large cohort studies, this model demonstrated superior predictive performance compared with traditional models and the WHO classification. It applies to SFT patients undergoing R0 resection in both central and extra-central nervous system locations, thereby overcoming the limitations of previous models (37). Combined with the patient’s complete clinical and pathological indicators (34-year-old male, maximum tumor diameter of 10.4 cm, mitotic count <4/10 HPF, absence of tumor necrosis), individualizzed risk evaluation was conducted using the mDemicco risk model. This case scored 3 points and was stratified into the low-risk category. Despite its low-risk classification, SFT exhibits inherent biological heterogeneity and a persistent risk of long-term recurrence. Thus, intensive, long-term follow-up is still required to detect early tumor recurrence.

**Table 1:** Risk stratification models for SFT.

Risk model	Year	Core evaluated indicators	Risk stratification	Stratification criteria	Primary endpoints	Target population	Reference
Original Demicco model	2012	Age, tumor size, mitotic count	Low, Intermediate, High	Total score 0–2: Low; 3–4: Intermediate; 5–6: High. Age: <55 years = 0, ≥55 years = 1; Tumor size: <5 cm = 0, 5 to <10 cm = 1, 10 to <15 cm = 2, ≥15 cm = 3; Mitotic count: 0/10 HPF = 0, 1–3/10 HPF = 1, ≥4/10 HPF = 2	Metastasis, disease-specific survival	Extracranial SFT	(33)
Salas overall survival model (SalasOS)	2017	Age, mitotic count	Low, Intermediate, High	Low: <60 years and mitotic count ≤4/10 HPF; Intermediate: 1 risk factor; High: ≥60 years and mitotic count >4/10 HPF	Overall survival, metastatic risk	Extracranial SFT	(34)
Modified Demicco model (mDemicco)	2019	Age, tumor size, mitotic count, tumor necrosis	Low, Intermediate, High	Comprehensive scoring of four variables; total score 0–3: Low; 4-5: Intermediate; 6-7: High; tumor necrosis ≥10% as an additional high-risk factor	Disease-free survival, metastasis, local recurrence	Extracranial SFT	(35)
G-score model	2022	Mitotic count, tumor necrosis, sex	Low, Intermediate, High	Low: Female, mitotic count <4/10 HPF, no necrosis; Intermediate: 1–2 low-risk criteria; High: Male, mitotic count ≥4/10 HPF, presence of necrosis	Early/late recurrence, disease-free survival	Extracranial SFT	(36)
Integrated risk model	2023	Mitotic count, Ki-67+ cell density, CD163+ cell density, MTOR mutation	Low, Intermediate, High	Low: total score = 0; Intermediate: 0 < total score ≤ 3.37. High: total score > 3.37. Mitotic count ≥4/10 HPF = 2.42; MTOR mutation = 4.56; Ki-67+ ≥454.7 cells/mm² = 0.949; CD163+ ≥929.3 cells/mm² = 2.07	Progression-free survival (PFS), recu rence, metastasis	Primary non-CNS and CNS SFT with negative tumor margins (R0)	(37)

SFT, solitary fibrous tumor; HPF, high-power field; CNS, central nervous system.

In summary, this study is a single-case report with a small sample size and only 1 year of follow-up. Therefore, the long-term safety and efficacy of robot-assisted laparoscopic surgery for giant pelvic SFT cannot be fully confirmed. Further accumulation of cases and multicenter prospective clinical studies are needed to clarify therapeutic strategies and prognostic factors across different SFT subtypes. The preoperative diagnosis, surgical planning, and long-term follow-up management should be continuously optimized to promote the application of precision medicine in the clinical diagnosis and treatment of SFT.

## 4 Conclusion

For appropriately selected patients, robot-assisted laparoscopic resection can serve as a feasible minimally invasive strategy for giant pelvic SFT. This approach helps achieve meticulous dissection and R0 resection, with favorable short-term outcomes. Given the biological heterogeneity of SFT and its risk of late recurrence, regular long-term follow-up is strongly recommended for affected patients.

## Data Availability

The original contributions presented in the study are included in the article/supplementary material. Further inquiries can be directed to the corresponding author.
